# The role of 14,15-dihydroxyeicosatrienoic acid levels in inflammation and its relationship to lipoproteins

**DOI:** 10.1186/1476-511X-12-151

**Published:** 2013-10-23

**Authors:** Tian Yang, Ran Peng, Yuan Guo, Li Shen, Shuiping Zhao, Danyan Xu

**Affiliations:** 1 Department of Cardiology, Internet Medicine, The Second Xiangya Hospital, Central South University, Changsha 410011, Hunan, China

**Keywords:** Coronary heart disease, Atherosclerosis, 14,15-epoxyeicosatrienoic acids, 14,15-dihydroxyeicosatrienoic acids, Inflammation, Lipoproteins

## Abstract

**Background:**

14,15-Epoxyeicosatrienoic acids (14,15-EETs) generated from arachidonic acid by cytochrome P450 epoxygenases have beneficial effects in certain cardiovascular diseases, and increased 14,15-EET levels protect the cardiovascular system. 14,15-EETs are rapidly hydrolyzed by soluble epoxide hydrolase (sEH) to the corresponding 14,15-dihydroxyeicosatrienoic acids (14,15-DHETs), which are generally less biologically active but more stable metabolite. A functionally relevant polymorphism of the CYP2J2 gene is independently associated with an increased risk of coronary heart disease (CHD), and the major CYP2J2 product is 14,15-EETs. 14,15-DHETs can be considered a relevant marker of CYP2J2 activity. Therefore, the aim of the present study was to evaluate the plasma 14,15-DHET levels to reflect the 14,15-EET levels in an indirectly way in patients with CHD, and to highlight the growing body of evidence that 14,15-EETs also play a role in anti-inflammatory and lipid-regulating effects in patients with CHD. This was achieved by investigating the relationship between 14,15-DHETs and high-sensitivity C-reactive protein (hs-CRP) and blood lipoproteins.

**Methods:**

Samples of peripheral venous blood were drawn from 60 patients with CHD and 60 healthy controls. A 14,15-DHET enzyme-linked immunosorbent assay kit (14,15-DHET ELISA kit) was used to measure the plasma 14,15-DHET levels. Hs-CRP, total cholesterol, triglyceride, high-density lipoprotein cholesterol, and low-density lipoprotein-cholesterol levels were measured.

**Results:**

14,15-DHET levels (2.53 ± 1.60 ng/mL) were significantly higher in patients with CHD as compared with those of the healthy controls (1.65 ± 1.54 ng/mL, P < 0.05). There was a significant positive correlation between 14,15-DHETs and hs-CRP levels (R = 0.286, P = 0.027). However, there was no significant correlation between 14,15-DHETs and blood lipoproteins (all, P > 0.05).

**Conclusions:**

Increased plasma 14,15-DHET levels reflect the decreased of 14,15-EET levels in an indirectly way. Indicated that decreased plasma 14,15-EET levels might be involved in the inflammatory reaction process in atherosclerosis.

## Introduction

Over the past decade, it has become increasingly apparent that epoxyeicosatrienoic acids (EETs) have cardiovascular protective effects, including vasodilation, angiogenesis, decreasing platelet aggregation, and generally acting to maintain vascular homeostasis. More importantly, EETs have anti-inflammatory effects that play an important role in the prevention of coronary heart disease (CHD) [1-4]. EETs are hydrolyzed by soluble epoxide hydrolase (sEH) to the corresponding dihydroxyeicosatrienoic acids(DHETs); thus, it is expected that the inhibition of this enzyme enhances the beneficial cardiovascular properties of EETs [5]. Therefore, sEH inhibitors (sEHIs) have been rapidly developed and have been proven beneficial in cardiovascular diseases such as hypertension and CHD [5,6].

It is well known that inflammation plays a very important role in the development and prognosis of CHD. The initial findings of the anti-inflammatory properties of EETs described by Node et al. [7] that EETs inhibited the activation of nuclear factor kappa B (NF-κB), a key transcription factor involved in the expression of numerous pro-inflammatory genes. EETs were also found to inhibit the expression of vascular cell adhesion molecule-1 in human endothelial cells in response to tumor necrosis factor-alpha, interleukin-1 alpha, or lipopolysaccharide [7]. Some studies [8] have demonstrated that peroxisome proliferator–activated receptor gamma (PPARγ) activation contributes to the anti-inflammatory effects of cytochrome P450 (CYP)-derived EETs. A number of studies [9-11] have also demonstrated that CYP-derived EETs inhibit cyclooxygenase 2–mediated inflammatory responses. Therefore, EETs might be an indicator reflecting the state of inflammation. But EETs are very unstable metabolites, it's rapidly hydrolyzed by sEH to the less biologically active but more stable metabolites DHETs. Spiecker et al. [12] demonstrated that a functionally relevant polymorphism of the CYP2J2 gene is independently associated with an increased risk of CHD, and the major CYP2J2 product is 14,15-EETs. 14,15-DHETs can be considered a relevant marker of CYP2J2 activity. Therefore, in our study we chosen 14,15-DHETs to reflect the 14,15-EET levels in an indirectly way in patients with CHD.

From another point of view, high-sensitivity C-reactive protein (hs-CRP), an acute-phase reactive protein, is now recognized as a good indicator of inflammation and a pro-inflammatory atherogenic circulating marker that has been proven an independent cardiac risk predictor [13]. It has been shown that hs-CRP is of prognostic value in patients with acute coronary syndromes and plays an important role in the development of CHD [14]. However, the relationship between EETs and hs-CRP remains unclear.

Dyslipidemia also is an independent risk factor in the progress of CHD [15,16]. Low-density lipoprotein cholesterol (LDL-C) is atherogenic and represents a strong cardiovascular risk factor [17]. High-density lipoprotein cholesterol (HDL-C) mediates reverse cholesterol transport and exerts several atheroprotective effects [18]. Epidemiologic evidence has shown that low HDL-C is a strong and independent cardiovascular risk marker [19].

Some research has found that local adipose tissue inflammation and inflammatory lipid mediators, including EETs, may play important roles in regulating adipocyte function and lipid metabolism [20] and EETs can activate PPARγ, which may play important roles in lipid metabolism [21]. However, the specific mechanism is still unknown; hence, we investigated the relationship between 14,15-DHETs and blood lipoproteins to determine whether they are related.

To date, there are no reports about the relationship between 14,15-EETs and hs-CRP and blood lipoprotein in patients with CHD. In order to explore the beneficial effects of EETs further, we studied the plasma 14,15-DHET levels in patients with CHD, and investigated the relationship between 14,15-DHETs and hs-CRP as well as blood lipoproteins in these patients.

## Materials and methods

### Subjects

We recruited 120 people for this study. We enrolled 60 CHD patients (44 men and 16 women) ranging in age from 51 to 69 years old (average age 60.42 ± 8.75 years) in the study, which spanned September 2010 to December 2012 in our hospital. We recruited 60 age- and sex-matched healthy subjects as controls (42 men and 18 women, average age 61.09 ± 8.87 years).

The diagnosis of CHD was defined as having experienced symptoms of myocardial ischemia such as angina, and stenosis in at least one major epicardial coronary artery by coronary angiography. All participants were clinically stable and chest pain free at the time of their study visit. Patients with the following diseases and situations were excluded from the CHD group: left ventricular systolic dysfunction (ejection fraction ≤ 35%), current use of insulin, active autoimmune disease, history of severe aortic stenosis, history of solid organ transplant or dialysis, or history of cancer within the previous five years. A detailed medical and medication history and fasting serum chemistry and cholesterol panel were obtained from the healthy volunteers; individuals with a history of cardiovascular disease or risk factors for coronary artery disease were excluded.

The Second Xiangya Hospital of Central South University Ethics Committee approved the study protocol. Blood was collected by venipuncture. Plasma was separated by centrifugation and stored at -80°C pending analysis.

### Measurements and methods

We collected 120 samples of peripheral venous blood, and then separated the plasma supernatant by centrifugation. An enzyme-linked immunosorbent assay (ELISA) was used to measure the plasma 14,15-DHET (14,15-DHET ELISA kit; Detroit R&D Inc., Detroit, MI, USA) according to the manual. A specialist who was unaware of the subjects’ assignations analyzed the plasma hs-CRP, total cholesterol (TC), triglyceride (TG), HDL-C, LDL-C, and biochemical indicators of liver and kidney function using a Hitachi 7170A analyzer (Hitachi, Tokyo, Japan).

### Statistical analysis

All data were analyzed with SPSS 16.0. Continuous variables between groups were analyzed by an independent sample t-test. Correlations were tested by the Spearman rank correlation coefficient. Differences were considered significant if the null hypothesis could be rejected with >95% confidence. P-values < 0.05 (two-tailed) were considered to indicate statistical significance. The log-transformed hs-CRP and other values are presented as mean ± standard error (mean ± SE).

## Results

### Characteristics of study participants

TG and LDL-C levels were higher in the CHD patients than in the control group (P < 0.05), but the HDL-C levels of the CHD patients tended to be lower (P < 0.05). No significant difference was observed for other indicators such as age, sex, body mass index, smoking status, TC, fasting blood glucose, blood urea nitrogen, serum creatinine, alanine aminotransferase, and aspartate aminotransferase (all, P > 0.05; Table 1).

**Table 1:** Demographic and biochemical characteristics of study participants

	**CHD patients**	**Healthy controls**	**P**
	**n=60**	**n=60**	
Age (years)	60.42±8.75	61.09±8.87	NS
Male/Female	44/16	42/18	NS
BMI (kg/m^2^)	24.59±3.36	24.63±2.91	NS
Smoking cases (yes/no)	38/22	36/24	NS
TG (mmol/L)	1.75±0.91	1.33±0.68	0.041^a^
TC (mmol/L)	4.48±1.01	4.19±0.92	NS
HDL-C (mmol/L)	1.04±0.29	1.20±0.28	0.018^a^
LDL-C (mmol/L)	2.78±1.10	2.24±0.71	0.036^a^
FBS (mmol/L)	6.49±3.06	5.94±2.79	NS
BUN (mmol/L)	5.51±1.80	5.67±1.40	NS
Cr (umol/L)	81.61±19.33	78.85±16.87	NS
ALT (mmol/L)	24.68±9.99	20.58±8.21	NS
AST (mmol/L)	26.79±9.45	24.24±7.44	NS
ACE inhibitor or ARB (%)	36 (60%)	0 (0%)	
Beta-blocker (%)	49 (82%)	0 (0%)	
Statin (%)	60 (100%)	0 (0%)	
Aspirin (%)	60 (100%)	0 (0%)	
Clopidogrel (%)	15 (25%)	0 (0%)	

^a^P < 0.05; CHD: Coronary heart disease; BMI: body mass index; TG: triglyceride; TC: total cholesterol; HDL-C: high-density lipoprotein cholesterol; LDL-C: low-density lipoprotein cholesterol; FBS: fasting blood glucose; BUN: blood urea nitrogen; Cr: serum creatinine; ALT: alanine aminotransferase; AST: aspartate aminotransferase; NS: not significant.

### 14,15-DHETs and hs-CRP levels between the two groups

**Figure 1:**
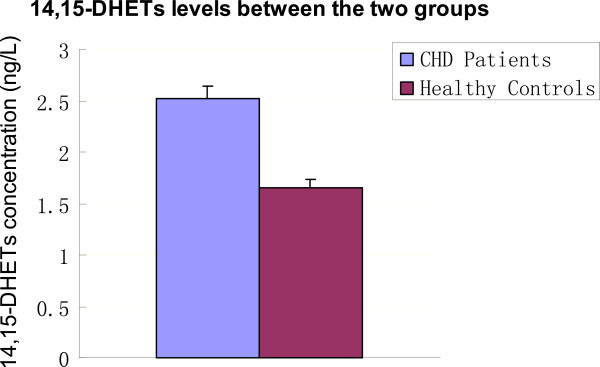
**14,15-DHET levels between the two groups.** CHD: Coronary heart disease; 14,15-DHETs: 14,15-dihydroxyeicosatrienoic acids.

**Figure 2:**
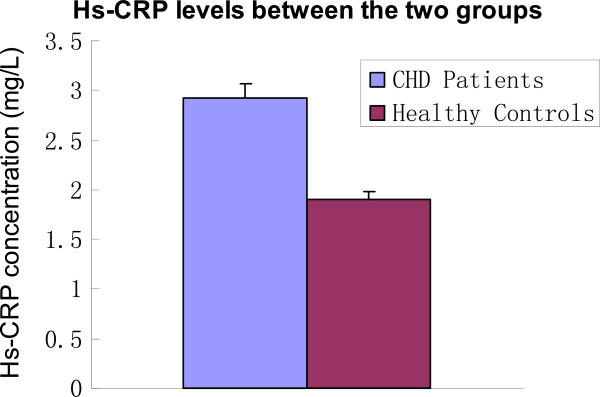
**Hs-CRP levels between the two groups.** CHD: Coronary heart disease; hs-CRP: high-sensitivity C-reactive protein.

As shown in Table 2, the 14,15-DHET levels in the CHD group were significantly higher than that in the control group (P < 0.05; Table 2; Figure 1), while hs-CRP levels were significantly higher in the CHD group (P < 0.01; Table 2; Figure 2).

**Table 2:** 14,15-DHETs and hs-CRP levels between the two groups

	**CHD patients**	**Healthy controls**	**P**
	**n = 60**	**n = 60**	
14,15-DHETS (ng/ml)	2.53 ±1.60	1.65 ±1.54	0.036^a^
hs-CRP (mg/L)	2.92±1.20	1.89±1.09	0.001^b^

^a^P < 0.05; ^b^P < 0.01; 14,15-DHETs: 14,15-dihydroxyeicosatrienoic acids; hs-CRP: high-sensitivity C-reactive protein.

### Correlation analysis of 14,15-DHETs and hs-CRP and blood lipoproteins

**Figure 3:**
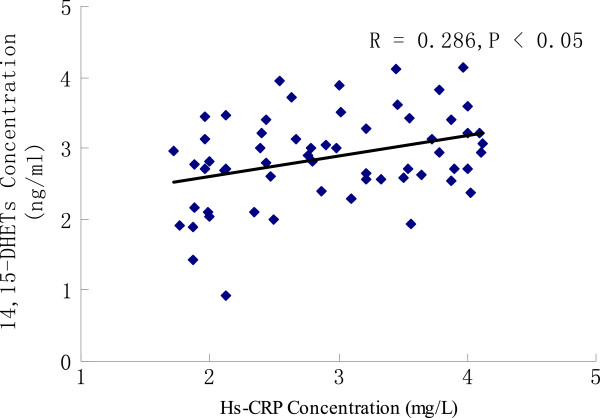
**Scatter plot of the correlations between 14,15-DHETs and hs-CRP in patients with CHD.** 14,15-DHETs: 14,15-dihydroxyeicosatrienoic acids. hs-CRP: high-sensitivity C-reactive protein. The scatter diagram depicts the 14,15-DHETs and hs-CRP concentrations. Spearman correlation analysis revealed a positive correlation between serum 14,15-DHETs and hs-CRP.

We performed correlation analysis to elucidate the relationship between 14,15-DHETs and hs-CRP and blood lipoproteins. There was a significant positive correlation between 14,15-DHET and hs-CRP levels (R = 0.286, P = 0.027). However, there did not appear to be a significant correlation between 14,15-DHETs and blood lipoproteins such as TC, TG, LDL-C, and HDL-C (P > 0.05; Table 3; Figure 3).

**Table 3:** Related coefficients of 14,15-DHETs and hs-CRP, TG, TC, LDL-C, and HDL-C in patients with CHD

	**hs-CRP**	**TG**	**TC**	**LDL-C**	**HDL-C**
14,15-DHETs	0.286	0.053	0.134	0.058	-0.005
	(P=0.027)^a^	(P=0.638)	(P=0.304)	(P=0.652)	(P=0.968)

^a^P < 0.05; 14,15-DHETs: 14,15-dihydroxyeicosatrienoic acids; hs-CRP: high-sensitivity C-reactive protein; TG: triglyceride; TC: total cholesterol; LDL-C: low-density lipoprotein cholesterol; HDL-C: high-density lipoprotein cholesterol.

## Discussion

In the present study, we found higher serum 14,15-DHETs, hs-CRP, TG, and LDL-C concentrations in patients with CHD as compared to the control group, but HDL-C levels were lower in patients with CHD. There was also a significant positive correlation between 14,15-DHET and hs-CRP levels. However, there was no specific relationship between 14,15-DHETs and blood lipoproteins.

The results of the present study show that the 14,15-DHET levels were significantly higher in patients with CHD. The process of CHD is closely related with coronary endothelial injury and dysfunction, and myocardial ischemia. Endothelial cells play important roles in vasculogenesis and re-endothelialization after ischemic injury. One of the functions of EET that has been explored is the ability of EETs to prevent apoptosis and promote the growth of endothelial cells [22-24]. A potent inhibitor of apoptosis, 14,15-EETs can serve as an intracellular second messenger for epidermal growth factor in cells expressing epoxygenase activity, and promote endothelial cell growth [23]. Yan et al. [25] found that specific epoxidation of EET sites produces endogenous PPARγ agonists, increasing cell proliferators, which might affect angiogenesis and cardiac recovery after ischemic infarct and reperfusion. Xu et al. [26] also demonstrated that sEHIs can promote angiogenesis by activating the EET–PPARγ pathway, which in turn increases vascular endothelial growth factor and hypoxia-inducible factor 1 alpha and triggers the migration and proliferation of endothelial progenitor cells. Numerous studies have suggested the beneficial effects of EETs on cardiac recovery following ischemia/reperfusion (I/R). Nithipatikom et al. [27] found that exogenous EETs produced a marked reduction in infarct size in dogs. Another study reported that the expression of CYP2J2 in cardiomyocytes led to improved functional recovery and reduced infarct size after ischemia [28]. EETs were reported to have aided in the preservation of mitochondrial integrity and membrane potential during I/R [29]. Similarly, EETs have been shown to release met-enkephalin, which binds δ-opioid receptors to reduce infarct size after I/R in rat heart [30]. EETs exert many other cardioprotective effects, including the reduction of myocardial stunning, myocardial infarct size, and inflammatory response; prevention of the onset of left ventricular hypertrophy and subsequent remodeling, which leads to heart failure; and reduction of the incidence of cardiac arrhythmias associated with heart failure [31]. Our findings are consistent with these results. But in Theken’s [32] study, CHD patients had higher EETs and tended to have lower DHETs compared to healthy volunteers, and significantly lower apparent sEH metabolic activity in the presence of stable atherosclerotic cardiovascular disease. These results are differ from us, we can not rule out the possibility that the detected difference will achieve statistical significance when future investigations study much larger patient groups. Even so, all the results lead up to the conclusion that EETs have a positive effect on the cardiovascular system.

We found that the plasma hs-CRP levels in patients with CHD were significantly higher than that in the control group. Inflammation is an important feature of atherosclerotic plaque [33,34]. As a vascular inflammatory marker, hs-CRP is closely related to cardiovascular disease. In patients with CHD, serum CRP levels gradually increase with the progress of the disease [35]. This is partially because CHD can cause ischemia and hypoxia, which leads to local tissue damage, myocardial infarction, myocardial fibrosis and necrosis, and neutrophil infiltration, thus stimulating the production of CRP. This clearly suggests that serum CRP levels are associated with the occurrence and development of CHD [35]. Schnell-Inderst et al. [13] found that the incidence of cardiovascular events was closely related to hs-CRP levels, and that hs-CRP can be used as an additional predictor in cardiovascular events.

We found a significant positive correlation between 14,15-DHET and hs-CRP levels in patients with CHD. This conclusion reflected that there might be a negative correlation between 14,15-EETs and hs-CRP in patients with CHD. Node et al. [36] found that EETs decreased cytokine-induced endothelial cell adhesion molecule expression and prevented leukocyte adhesion to the vascular wall by a mechanism involving the inhibition of the transcription factors NF-κB and inhibitor of kappa B kinase. The inhibitory effects of EETs were independent of their membrane-hyperpolarizing effects, suggesting that these molecules play an important nonvasodilatory role in vascular inflammation [36]. These findings and our results strongly suggest that EETs may be involved in the anti-atherosclerotic process by their inhibition of the inflammatory response. But in Schuck’s [37] research, no associations were observed between biomarkers of CYP-mediated eicosanoid metabolism and hs-CRP, it suggest that CYP-derived eicosanoids may be important in the regulation of vascular, but not hepatic or systemic, inflammation in humans. These results are differ from us, we can not rule out the possibility that the detected difference will achieve statistical significance, or the medication used causes different results.

We found much evidence from epidemiologic, clinical, and laboratory data indicating that elevated TG levels are an independent risk factor for cardiovascular disease [38,39]. However, we found no significant correlation between 14,15-DHETs and TC, TG, LDL-C, and HDL-C. It is worth mentioning that some studies have demonstrated that sEHIs have anti-atherosclerotic effects, and that the anti-atherosclerotic effects are correlated with elevation in EET levels and associated with LDL-C reduction and HDL-C elevation, as well as attenuation of the expression of pro-inflammatory genes and proteins [40-42]. Zhang et al. [40] demonstrated that sEH inhibition could lower circulating cholesterol levels, which could also contribute to the attenuation of atherosclerosis. In contrast, many studies have demonstrated that lipoproteins play a key role in precipitating CHD [43]. Moreover, some studies have suggested that in view of its molecular structures, sEH is involved in cholesterol, fatty acid, and lipid metabolism [44-46]. It is known that EETs are potent endogenous PPARα agonists, and as PPARα activation can increase HDL-C by increasing the concentration of apolipoproteins A-I and A-II and by stimulating the reverse cholesterol transport pathway [47] it is expected to affect blood lipoproteins. However, we did not find a significant correlation between 14,15-DHETs and blood lipoproteins. Pritchard et al. [48] found that endothelial cells incubated in atherogenic LDL concentrations produced substantially greater quantities of EET species. Karara et al. [49] also found that the lipoprotein fraction with the highest EET concentration was LDL, followed by HDL and very low–density lipoprotein cholesterol. Thus far, no evidence shows that EETs and blood lipoproteins are not correlated. Therefore, we cannot rule out the possibility that the detected difference will achieve statistical significance when future investigations study much larger patient groups.

This study tested the relationship between 14,15-DHETs and hs-CRP and blood lipoproteins in patients with CHD. The in vivo cross-sectional design of the study presents several limitations. First, the levels of sEH and its enzymatic activity could be different between groups, 14,15-EET, 14,15-DHET, leukotoxin, and leukotoxin diol are potential biomarkers for assessing sEH activity in clinical trial subjects, our further studies are necessary to enroll these indicators to figure out the differences between two groups. It must also be noted that the we did not separated smoker and non-smoker, but there were no significant difference in the number of smokers between two groups, so the measured results are comparable. In addition, our analysis compared a well-treated population of patients with advanced cardiovascular disease to healthy individuals with no risk factors for cardiovascular disease. Thus, multiple potential confounding factors may have influenced the differences in 14,15-DHETs, hs-CRP and blood lipoprotein. We cannot determine whether the observed differences are due to the presence of atherosclerotic disease, or a consequence of drug therapy. Since the effects of these established therapies on circulating CYP-derived eicosanoid levels, and specifically sEH expression and metabolic activity, in humans are unknown, further studies are necessary to quantify these effects. And the number of patients enrolled in the study was relatively small, which might cause bias. Therefore, to expand the samples is what we need to do in our further researches.

## Conclusion

In summary, the plasma 14,15-DHET levels in patients with CHD were significantly higher and were positive correlated with hs-CRP levels, suggesting that the decrease in 14,15-EET levels may be involved in the inflammatory reaction process in atherosclerosis. The correlation of 14,15-EETs and blood lipoproteins remains to be investigated.

## Consent

Written informed consent was obtained from the patient for the publication of this report and any accompanying images.

## Competing interests

The authors declare that they have no competing interests.

## Authors’ contributions

All the authors were involved in the design of this study. TY and LS substantially contributed to the design of the study, performing the experiment, analysis of data, and drafting the manuscript. RP carried out the concentration analysis. YG participated in sample selection. DYX and SPZ made contribution to design, analysis and revision of the manuscript. All the authors have read and approved the final version.
